# New Coleoptera records from New Brunswick, Canada: Histeridae

**DOI:** 10.3897/zookeys.179.2493

**Published:** 2012-04-04

**Authors:** Reginald P. Webster, Scott Makepeace, Ian DeMerchant, Jon D. Sweeney

**Affiliations:** 1 Natural Resources Canada, Canadian Forest Service - Atlantic Forestry Centre, 1350 Regent St., P.O. Box 4000, Fredericton, NB, Canada E3B 5P7; 2 Habitat Program, Fish and Wildlife Branch, New Brunswick Department of Natural Resources, P.O. Box 6000, Fredericton, NB, Canada E3B 5H1

**Keywords:** Histeridae, new records, Canada, New Brunswick

## Abstract

Eighteen species of Histeridae are newly reported from New Brunswick, Canada. This brings the total number of species known from New Brunswick to 42. Seven of these species, *Acritus exguus* (Erichson), *Euspilotus rossi* (Wenzel), *Hypocaccus fitchi* (Marseul), *Dendrophilus kiteleyi* Bousquet and Laplante, *Platysoma cylindricum* (Paykull), *Atholus sedecimstriatus* (Say), and *Margarinotus harrisii* (Kirby) are recorded from the Maritime provinces for the first time. Collection and bionomic data are presented for these species.

## Introduction

Bousquet and Laplante (2006) reviewed the Histeridae of Canada. Histeridae live in dung, carcasses, decaying vegetable matter, under bark, and in nests of mammals, birds, and ants (Bousquet and Laplante 2006). Most species are predaceous. Species living in decaying organic matter feed mainly on Diptera larvae, whereas those living under bark feed on eggs, larvae, and pupae of wood-boring beetles, and thus, members of this family are important ecologically (Bousquet and Laplante 2006). However, the biology of most of the Histeridae occurring in North America is still poorly known.

Bousquet and Laplante (2006) reported 135 species of Histeridae from Canada and 22 species from New Brunswick. Majka (2008) added another two species in his review of the Atlantic Canadian Histeridae. Here, we report another 18 species, bringing the total number of species known from the province to 42.

## Methods and conventions

The following records are based in part on specimens collected as part of a general survey by the first author to document the Coleoptera fauna of New Brunswick, a study to document the Coleoptera fauna of barred owl (*Strix varia* Barton) nests, and specimens obtained as by-catch in Lindgren 12-funnel traps (ConTech Inc., Delta, BC) during a study to develop a general attractant for the detection of invasive species of Cerambycidae.

### Collection methods

A variety of collection methods were employed to collect the specimens reported in this study. General details are outlined in Webster et al. (2009, Appendix). See Webster et al. (in press) for details of the methods used for deployment of Lindgren traps and sample collection. A significant number of Histeridae were collected from the nest contents of barred owls, which usually nest several meters or more above ground in tree cavities or in artificial nest boxes. Trees were climbed, and the entire nest contents (usually about 2–3 L) were removed (usually when chicks were present or shortly after the chicks had left the nest). Nest contents were replaced with wood chips similar to those normally used in nest boxes. Contents were hand sifted, and all beetles were removed from the samples. A detailed description of the habitat was recorded for all specimens collected during the Coleoptera survey. Locality and habitat data are presented exactly as recorded on labels for each specimen. This information, as well as additional collecting notes, is summarized and discussed in the collection and habitat data for each species.

### Distribution

Distribution maps, created using ArcMap and ArcGIS, are presented for each species from New Brunswick. Every species is cited with current distribution in Canada and Alaska, using abbreviations for the state, provinces, and territories. New provincial records are indicated in bold under Distribution in Canada and Alaska. The following abbreviations are used in the text:

**Table:** 

**AK**	Alaska	**MB**	Manitoba
**YT**	Yukon Territory	**ON**	Ontario
**NT**	Northwest Territories	**QC**	Quebec
**NU**	Nunavut	**NB**	New Brunswick
**BC**	British Columbia	**PE**	Prince Edward Island
**AB**	Alberta	**NS**	Nova Scotia
**SK**	Saskatchewan	**NF & LB**	Newfoundland and Labrador

Acronyms of collections examined and referred to in this study are as follows:

**AFC** Atlantic Forestry Centre, Natural Resources Canada, Canadian Forest Service, Fredericton, New Brunswick, Canada

**CNC** Canadian National Collection of Insects, Arachnids and Nematodes, Ottawa, Ontario, Canada

**NBM** New Brunswick Museum, Saint John, New Brunswick, Canada

**RWC** Reginald P. Webster Collection, Charters Settlement, New Brunswick, Canada

## Results

Eighteen species of Histeridae are newly reported from New Brunswick, bringing the total number of species known from the province to 42 (Table 1.). Seven species, *Acritus exguus* (Erichson), *Euspilotus rossi* (Wenzel), *Hypocaccus fitchi* (Marseul), *Dendrophilus kiteleyi* Bousquet and Laplante, *Platysoma cylindricum* (Paykull), *Atholus sedecimstriatus* (Say), and *Margarinotus harrisii* (Kirby) are newly recorded for the Maritime provinces of Canada.

**Table 1.** Species of Histeridae known from New Brunswick, Canada

Subfamily Abraeinae MacLeay
**Tribe Plegaderinae Portevin**
*Plegaderus confusus* Bousquet & Laplante
*Plegaderus sayi* Marseul
**Tribe Acritini Wenzel**
*Acritus exiguus* (Erichson)**
*Aeletes politus* (LeConte)
**Subfamily Saprininae Blanchard**
*Baeckmanniolus dimidiatipennis* (LeConte)
*Euspilotus assimilis* (Paykull)
*Euspilotus rossi* (Wenzel)**
*Geomysaprinus moniliatus* (Casey)
*Gnathoncus barbatus* Bousquet & Laplante*
*Gnathoncus communis* (Marseul)*
*Gnathoncus rotundatus* (Kugelann)
*Hypocaccus bigener* (LeConte)
*Hypocaccus fitchi* (Marseul)**
*Hypocaccus fraternus* (Say)
**Subfamily Dendrophilinae Reitter**
**Tribe Dendrophilini Reitter**
*Dendrophilus kiteleyi* Bousquet & Laplante**
*Dendrophilus punctatus* (Herbst)*
**Tribe Paromalini Reitter**
*Carcinops pumilo* (Erichson)
*Paromalus teres* LeConte
**Subfamily Histerinae Gyllenhal**
**Tribe Platysomatini Bickhardt**
*Platysoma coarctatum* J.E. LeConte
*Platysoma cylindricum* (Paykull)**
*Platysoma deficiens* (Casey)*
*Platysoma gracile* J.E. LeConte
*Platysoma leconti* Marseul*
**Tribe Histerini Gyllenhal**
*Atholus bimaculatus* (Linnaeus)
*Atholus perplexus* (J.L. LeConte)*
*Atholus sedecimstriatus* (Say)**
*Hister abbreviatus* Fabricius
*Hister curtatus* LeConte
*Hister furtivus* LeConte
*Margarinotus brunneus* (Fabricius)
*Margarinotus cognatus* (LeConte)*
*Margarinotus confusus* Wenzel*
*Margarinotus egregius* (Casey)*
*Margarinotus faedatus* (LeConte)
*Margarinotus harrisii* (Kirby)**
*Margarinotus hudsonicus* (Casey)
*Margarinotus immunis* (Erichson)
*Margarinotus interruptus* (de Beauvois)
*Margarinotus lecontei* Wenzel
*Margarinotus merdarius* (Hoffmann)*
*Margarinotus stygicus* (J.E. LeConte)*
*Psiloscelis planipes* (LeConte)

**Notes:** *New to province, **New to Maritime provinces.

### Species Accounts

All records are species newly recorded for New Brunswick, Canada. Species followed by ** are newly recorded from the Maritime provinces of Canada.

The classification of the Histeridae follows Bouchard et al. (2011).

### Histeridae Gyllenhal, 1808

**Subfamily Abraeinae MacLeay, 1819**

**Tribe Acritini Wenzel, 1944**

#### Acritus exiguus

(Erichson, 1834)**

http://species-id.net/wiki/Acritus_exiguus

Map 1

##### Material examined.

**New Brunswick, York Co.**, 15 km W of Tracy off Rt. 645, 45.6848°N, 66.8821°W, 16–30.VI.2010, R. Webster & C. MacKay, old red pine forest, Lindgren funnel trap (1, RWC).

**Map 1.**
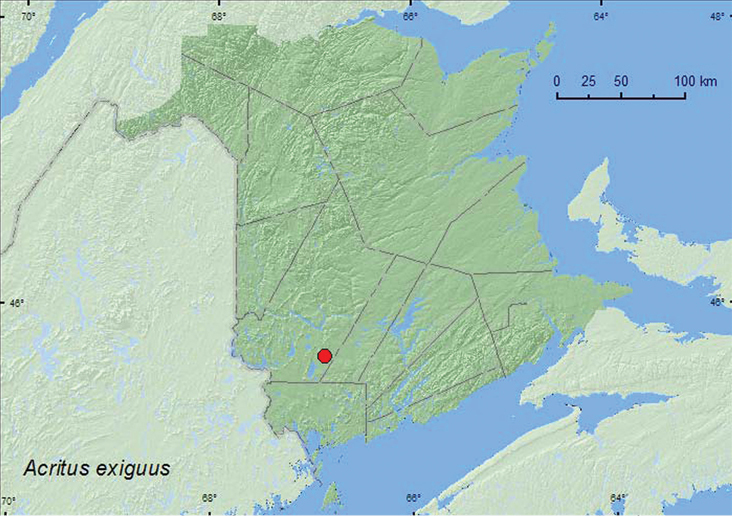
Collection localities in New Brunswick, Canada of *Acritus exiguus*.

##### Collection and habitat data.

This species occurs under bark of deciduous trees (Bousquet and Laplante 2006). The only specimen from New Brunswick was collected in a Lindgren funnel trap deployed in an old red pine (*Pinus resinosa* Ait.) forest. The adult was captured during June.

##### Distribution in Canada and Alaska

**.** ON, QC, **NB** (Bousquet and Laplante 2006).

### Subfamily Saprininae Blanchard, 1845

#### Euspilotus rossi

(Wenzel, 1939)**

http://species-id.net/wiki/Euspilotus_rossi

Map 2

##### Material examined. 

**New Brunswick, Queens Co.**, Rees near Grand Lake, 46.0016°N, 65.9466°W, 29.V.2007, S. Makepeace & R. Webster, in barred owl nest in an artificial nest box (2, CNC, RWC).

**Map 2.**
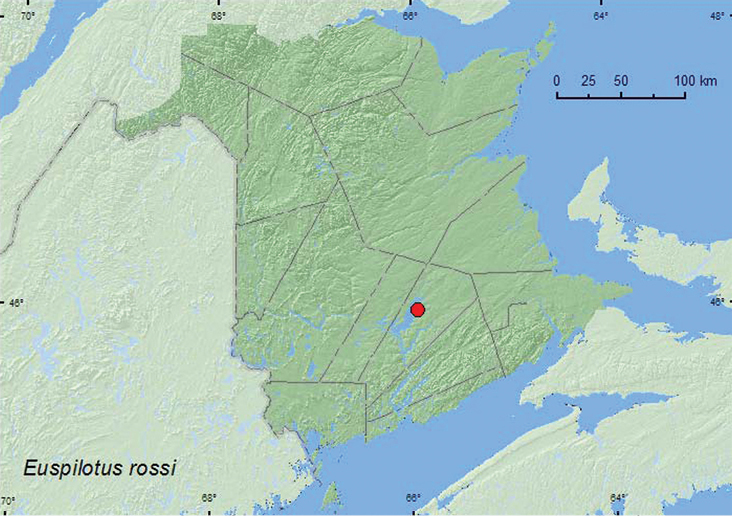
Collection localities in New Brunswick, Canada of *Euspilotus rossi*.

##### Collection and habitat data

**.** This species was reported from flicker (*Colaptes auratus* L.) nests by Kovarik and Caterino (2001). The specimens from New Brunswick were found in nest material of a barred owl nest with chicks. *Euspilotus rossi* was found in only one of 23 barred owl nests sampled (Webster and Makepeace, unpublished data), suggesting that this is not the usual habitat for this species. The two adults were collected in late May.

##### Distribution in Canada and Alaska.

ON, QC, **NB** (Bousquet and Laplante 2006). Only a few specimens of this rare species are known from Canada (Bousquet and Laplante 2006).

#### Gnathoncus barbatus

Bousquet & Laplante, 1999

http://species-id.net/wiki/Gnathoncus_barbatus

Map 3

##### Material examined. 

**New Brunswick, Carleton Co.,** Benton, 45.9961°N, 67.5864°W, 24.V.2007, Makepeace & R. Webster, in barred owl nest in natural tree cavity (1, RWC). **Queens Co.,** Pleasant Villa, 45.7023°N, 66.1732°W, 15.VI.2007, S. Makepeace & R. Webster, in barred owl nest in natural tree cavity (4, RWC, NBM); McAlpines near Upper Hampstead Rd., 45.7250°N, 66.1200°W, 3.VI.2007, S. Makepeace & R. Webster, in barred owl nest in natural tree cavity (4, RWC, NBM); Rees, near Grand Lake, 46.0016°N, 65.9466°W, 29.V.2007, S. Makepeace & R. Webster, in nest contents of barred owl in an artificial nest box (8, NBM). **Sunbury Co.**, Noonan, 45.9923°N, 66.4099°W, 2.VI.2007, S. Makepeace & R. Webster, in barred owl from tree hole 7 m high in red maple, damp organic material with small bones (1, NBM);Acadia Research Forest, 45.9866°N, 66.3841°W, 2–9.VI.2009, R. Webster & M.-A. Giguère, mature (100 year-old) red spruce forest with scattered red maple and balsam fir, Lindgren funnel trap (1, AFC). **Westmorland Co.**, Sackville near Ogden Mill, 45.9216°N, 64.3893°W, 12.V.2006, S. Makepeace & R. Webster, in great horned owl nest (2, RWC, NBM). **York Co.**, Charters Settlement, 45.8428°N, 66.7278°W, in decayed mushrooms, 16.IX.2004, R. P. Webster (1, RWC); Keswick Ridge, 46.0040°N, 66.8776°W, 23.V.2006, S. Makepeace & R. Webster, in barred owl nest in natural tree cavity (2, RWC, NBM); Pokiok Settlement (String Bog), 45.9101°N, 67.1235°W, 26.VI.2007, S. Makepeace & R. Webster, in barred owl nest in natural tree cavity (1, RWC); Marysville, 45.9750°N, 66.5700°W, 22.VI.2007, S. Makepeace & R. Webster, in barred owl nest, with dry organic material and remains of squirrel, birds, and insect parts (4, RWC, NBM).

**Map 3.**
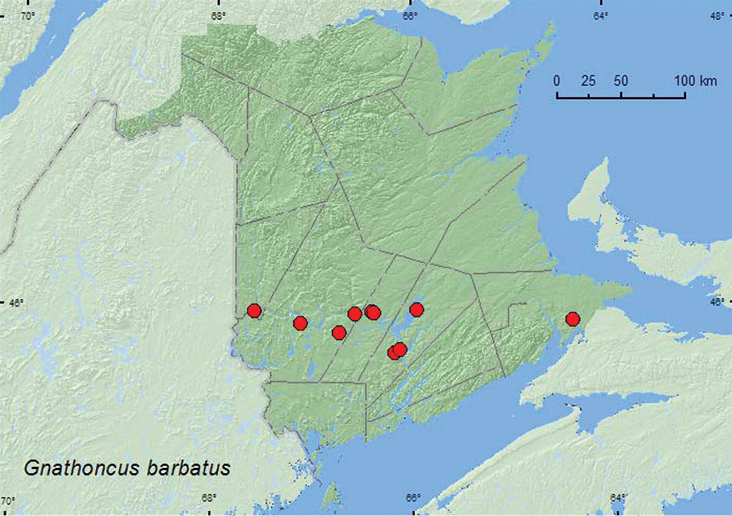
Collection localities in New Brunswick, Canada of *Gnathoncus barbatus*.

##### Collection and habitat data.

Little information was previously known about the habitat and biology of this species. Bousquet and Laplante (2006) reported one specimen from porcupine (*Erethizon dorsatum* (L.)) dung piled in a cavity at the base of an old sugar maple (*Acer saccharum* Marsh.). Most specimens from New Brunswick were collected from nest material of barred owl nests (most with chicks) in natural cavities in trees or in artificial nest boxes. Others were collected from a great horned owl (*Bubo virginianus* Gmelin) nest, decaying mushrooms, and a Lindgren funnel trap. This species is probably associated with birds and possibly mammals that nest in tree cavities. The nest contents of the barred owls and great horned owl contained decaying animal remains, and often many Diptera larvae were present on which the predaceous histerid adults and larvae were probably feeding. Adults were collected during May, June, and September.

##### Distribution in Canada and Alaska.

BC, AB, ON, QC, **NB**, NS (Bousquet and Laplante 2006).

#### Gnathoncus communis

(Marseul, 1862)

http://species-id.net/wiki/Gnathoncus_communis

Map 4

##### Material examined. 

**New Brunswick, Kings Co.**, near Quarries, 45.6005°N, 66.0500°W, 25.IX.2005, S. Makepeace & R. Webster, in barred owl nest in nest box on red maple, dry litter (2, RWC). **Queens Co.**, Elm Hill, 45.7140°N, 66.1315°W, 27.VI.2007, S. Makepeace & R. Webster, in barred owl nest in tree hole in red oak, damp (urine smell) organic material with feathers, fur and small bones (2, RWC).

**Map 4.**
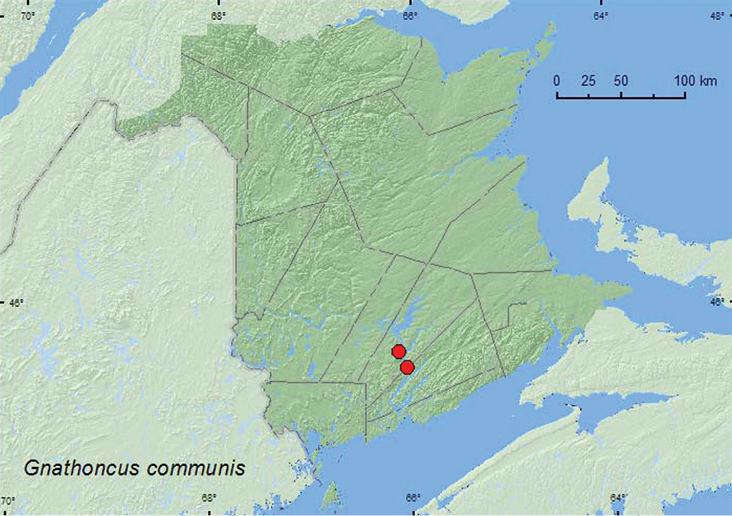
Collection localities in New Brunswick, Canada of *Gnathoncus communis*.

##### Collection and habitat data.

This species was collected from nest material from barred owl nests in natural cavities in trees or artificial nest boxes. Bousquet and Laplante (2006) reported this species as occurring in similar habitats. Adults were collected during June and September.

##### Distribution in Canada and Alaska.

BC, AB, MB, ON, QC, **NB**, NS (Bousquet and Laplante 2006). This species is possibly adventive in North America (Bousquet and Laplante 2006).

#### Hypocaccus fitchi

(Marseul, 1862)**

http://species-id.net/wiki/Hypocaccus_fitchi

Map 5

##### Material examined. 

**New Brunswick, Queens Co.**, Bayard, at Nerepis River, 45.4473°N, 66.3318°W, 24.V.2009, R. P. Webster, river margin, on sand bar in debris on sand (2, RWC).

**Map 5.**
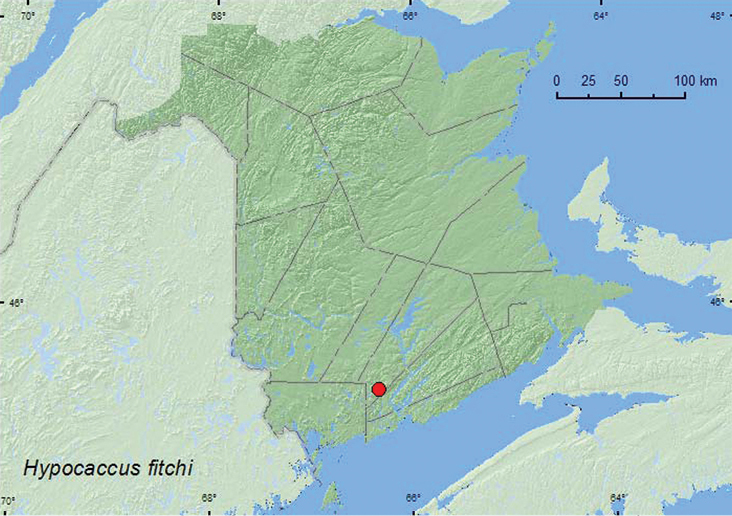
Collection localities in New Brunswick, Canada of *Hypocaccus fitchi*.

##### Collection and habitat data.

A few specimens from Quebec were found on sandy beaches along rivers (Bousquet and Laplante 2006). The New Brunswick specimens were found in debris resting on sand on a sand bar along a small river during late May.

##### Distribution in Canada and Alaska.

ON, QC, **NB** (Bousquet and Laplante 2006).

### Subfamily Dendrophilinae Reitter, 1909

**Tribe Dendrophilini Reitter, 1909**

#### Dendrophilus kiteleyi

Bousquet & Laplante, 1999**

http://species-id.net/wiki/Dendrophilus_kiteleyi

Map 6

##### Material examined. 

**New Brunswick, Kings Co.**, near Quarries, 45.6005°N, 66.0500°W, 25.IX.2005, S. Makepeace & R. Webster, in barred owl nest in nest box on red maple, dry litter (1, RWC). **Queens Co.**, Central Hampstead, 45.6575°N, 66.1412°W, 13.VII.2006, S. Makepeace & R. Webster, hardwood ridge, in nest of barred owl in tree hole (3, RWC, NBM); Elm Hill, 45.7140°N, 66.1315°W, 27.VI.2007, S. Makepeace & R. Webster, in barred owl nest in tree hole in red oak, damp (urine smell) organic material with feathers, fur and small bones (1, RWC); Pleasant Villa, 45.7023°N, 66.1732°W, 15.VI.2007, S. Makepeace & R. Webster, in barred owl nest in natural tree cavity (1, RWC); McAlpines near Upper Hampstead Rd., 45.7250°N, 66.1200°W, 3.VI.2007, S. Makepeace & R. Webster, in barred owl nest in natural tree cavity (4, RWC, NBM); Rees near Grand Lake, 46.0016°N, 65.9466°W, 29.V.2007, S. Makepeace & R. Webster, in nest contents of barred owl in artificial nest box (1, RWC). **York Co.,** Marysville, 45.9750°N, 66.5700°W, 22.VI.2007, S. Makepeace & R. Webster, in barred owl nest, with dry organic material and remains of squirrel, birds, and insect parts (1, RWC).

**Map 6.**
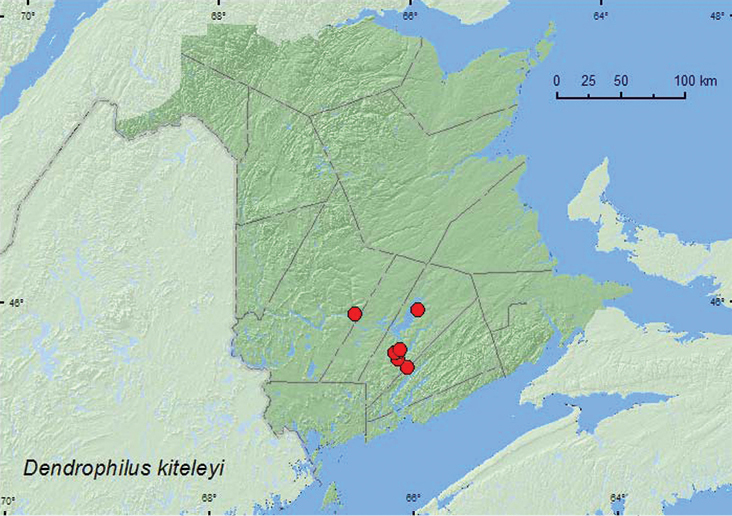
Collection localities in New Brunswick, Canada of *Dendrophilus kiteleyi*.

##### Collection and habitat data.

Most specimens of *Dendrophilus kiteleyi* were found in tree cavities in deciduous trees (Bousquet and Laplante 2006). All specimens from New Brunswick were collected from nest material from barred owl nests (most with chicks) in natural cavities in trees or in artificial nest boxes. Adults were collected during late May, June, July, and September.

##### Distribution in Canada and Alaska.

AB, MB, ON, QC, **NB** (Bousquet and Laplante 2006).

#### Dendrophilus punctatus

(Herbst, 1792)

http://species-id.net/wiki/Dendrophilus_punctatus

Map 7

##### Material examined. 

**New Brunswick, Queens Co.**, Elm Hill, 45.7140°N, 66.1315°W, 27.VI.2007, S. Makepeace & R. Webster, in barred owl nest with chicks in a natural cavity in a red oak (1, RWC).

**Map 7.**
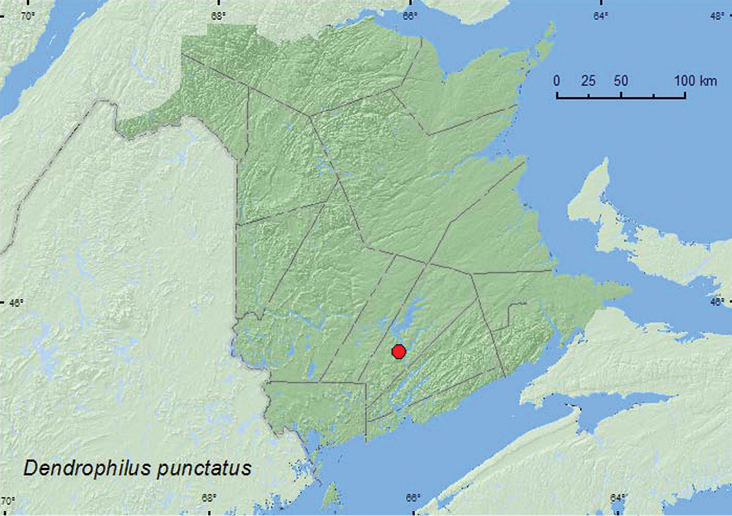
Collection localities in New Brunswick, Canada of *Dendrophilus punctatus*.

##### Collection and habitat data.

In Europe, this species was frequently found in bird nests, granaries, and mills (Hinton 1945). In Canada, most records of this species were from tree cavities in deciduous trees (Bousquet and Laplante 2006). The specimen from New Brunswick was taken from the nest material of a barred owl in a tree cavity in a red oak (*Quercus rubra* L.) during late June.

##### Distribution in Canada and Alaska.

MB, ON, QC, **NB**, NS (Bousquet and Laplante 2006). This adventive species is now widespread in North America (Bousquet and Laplante 2006).

### Subfamily Histerinae Gyllenhal, 1808

**Tribe Platysomatini Bickhardt, 1914**

#### Platysoma cylindricum

(Paykull, 1811)**

http://species-id.net/wiki/Platysoma_cylindricum

Map 8

##### Material examined. 

**New Brunswick, York Co.**, Fredericton, 28.VI.1929, L. J. Simpson, in tunnel of *Ips pini* (in pine) (1, AFC).

**Map 8.**
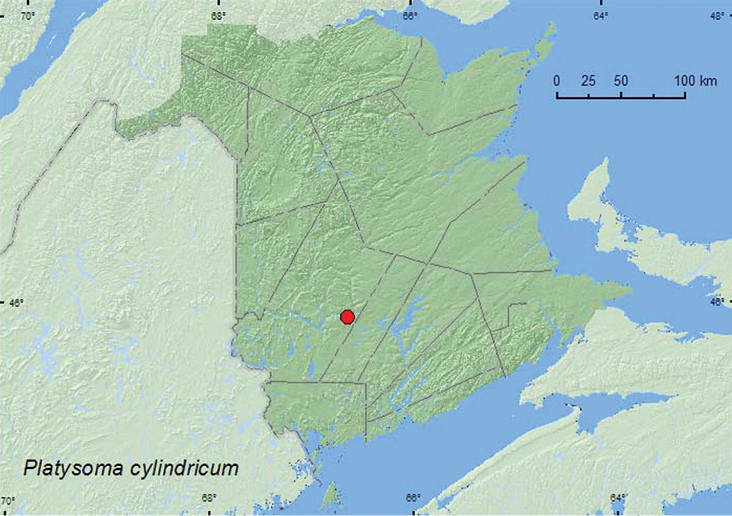
Collection localities in New Brunswick, Canada of *Platysoma cylindricum*.

##### Collection and habitat data.

This species occurs under bark of dead pines (Bousquet and Laplante 2006). The New Brunswick specimen was found in a tunnel of *Ips pini* (Say), presumably in a dead pine. The adult was captured during late June.

##### Distribution in Canada and Alaska.

ON, QC, **NB** (Bousquet and Laplante 2006).

#### Platysoma deficiens

(Casey, 1924)

http://species-id.net/wiki/Platysoma_deficiens

Map 9

##### Material examined. 

**New Brunswick, Charlotte Co.**, Oak Bay, 6.VII.1928, L. J. Simpson, from *Ips pini* tunnels (1, AFC). **Sunbury Co.**, Acadia Research Forest, 45.9866°N, 66.3841°W, 18–24.VI.2009, 24-30.VI.2009, 18-31.VIII.2009, R. Webster & M.-A. Giguère, mature (110 year-old) red spruce forest with scattered red maple and balsam fir, Lindgren funnel traps (3, AFC, RWC). **York Co.**, Taymouth, 29.VI.1929 (no collector given) (1, AFC); Fredericton, 22.VI.1929, L. J. Simpson, (1, AFC); 15 km W of Tracy off Rt. 645, 45.6848°N, 66.8821°W, 21-28.VI.2009, 7-14.VII.2009, 4-11.VIII.2009, 11-18.VIII.2009, R. Webster & M.-A. Giguère, old red pine forest, Lindgren funnel traps (4, AFC, RWC); same locality data but 6.VI.2009, R. Webster & M.-A. Giguère, old red pine forest, under bark scales of recently fallen red pine (4, RWC); 14 km WSW of Tracy, S of Rt. 645, 45.6741°N, 66.8661°W, 10-26.V.2010, R. Webster & C. MacKay, old mixed forest with red and white spruce, red and white pine, balsam fir, eastern white cedar, red maple, and *Populus* sp., Lindgren funnel traps (6, AFC).

**Map 9.**
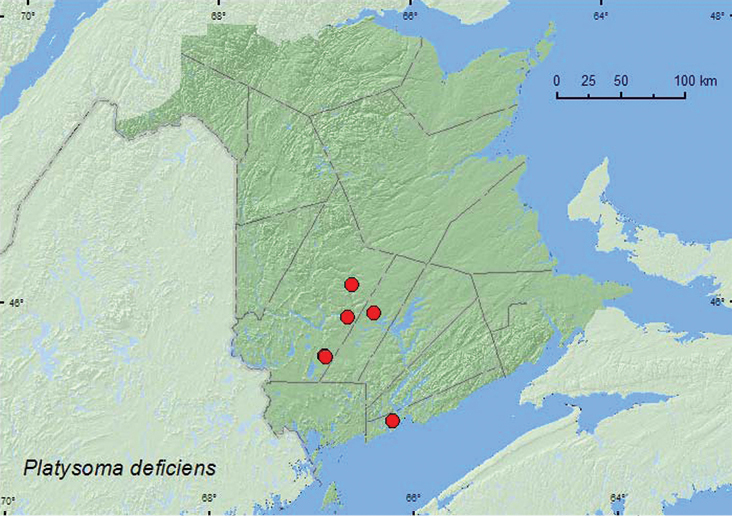
Collection localities in New Brunswick, Canada of *Platysoma deficiens*.

##### Collection and habitat data.

This species is found under bark of dead pines (*Pinus* spp.), spruce (*Picea* spp.), and larches (*Larix* sp.) (Bousquet and Laplante 2006). In New Brunswick, specimens were collected from *Ips pini* tunnels, from under bark scales of a recently fallen red pine, and in Lindgren funnel traps in a variety of forest types with conifers. Adults were collected during May, June, July, and August.

##### Distribution in Canada and Alaska.

AB, SK, MB, ON, QC, **NB**, NS (Bousquet and Laplante 2006).

#### Platysoma leconti

Marseul, 1853

http://species-id.net/wiki/Platysoma_leconti

Map 10

##### Material examined. 

**New Brunswick, Carleton Co.,** Meduxnekeag Valley Nature Preserve, 46.1888°N, 67.6762°W, 20.V.2005, R. P. Webster (1, RWC). **Northumberland Co.**, 12.0 km SSE of Upper Napan near Goodfellow Brook, 46.8943°N, 65.3810°W, 23.V.2007, R. P. Webster, recent clear-cut, under bark of spruce log (1, NBM). **Queens Co.**, Cranberry Lake P.N.A. (Protected Natural Area), 46.1125°N, 65.6075°W, 25.VI.2009, R. Webster & M.-A. Giguère, mature red oak forest, on dead red oak trunk (1, AFC); same locality data and forest type, 13–25.V.2011, 29.VI–7.VII.2011, M. Roy & V. Webster, Lindgren funnel traps (2, NBM). **Restigouche Co.**, Jacquet River Gorge P.N.A., 47.8779°N, 66.0013°W, 13.VI.2009, R. P. Webster, mixed forest, under bark of birch with fermented sap (2, RWC). **Sunbury Co.**, Portobello Creek N.W.A., 45.8992°N, 66.4248°W, 5.VI.2004, R. P. Webster (1, RWC); Lakeville Corner, 45.9007°N, 66.2423°W, 27.VIII.2006, R. P. Webster, silver maple swamp, among polypore fungi on poplar log (1, NBM). **York Co.**, near Magaguadavic Lake, 45.7283°N, 67.1818°W, 24.IV.2004, D. Sabine & R. Webster (3, NBM, RWC); Charters Settlement, 45.8340°N, 66.7450°W, 14.V.2004, R. P. Webster (1, RWC); 15 km W of Tracy off Rt. 645, 45.6848°N, 66.8821°W, 4–11.VIII.2009, R. Webster & M.-A. Giguère, old red pine forest, Lindgren funnel trap (1, AFC).

**Map 10.**
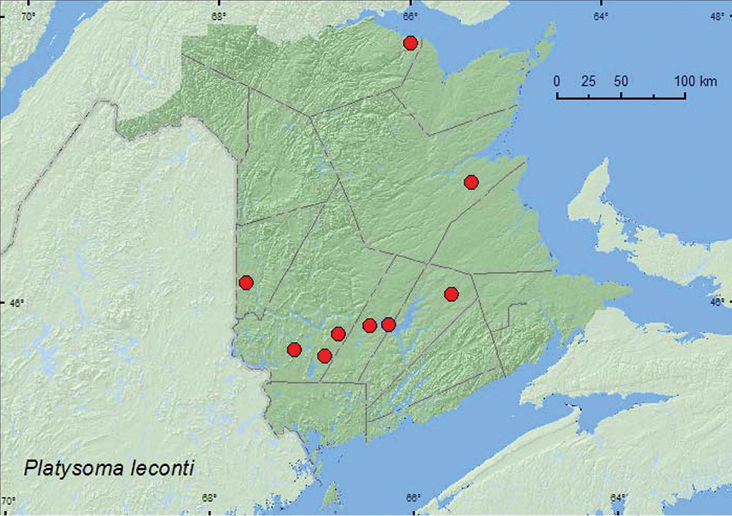
Collection localities in New Brunswick, Canada of *Platysoma leconti*.

##### Collection and habitat data.

*Platysoma leconti* is found under bark of a variety of dead deciduous tree species (maples (*Acer* spp.), oaks (*Quercus* spp.), elm (*Ulmus* spp.), poplars (*Populus* sp.), ashes (*Fraxinus* spp.)) and pines (Bousquet and Laplante 2006). In New Brunswick, this species was found under bark of a red oak, under bark with fermented sap of a dead standing birch (*Betula* sp.), and among polypore fungi on a poplar log. Adults were also caught in Lindgren funnel traps. Adults were captured during April, May, June, and August.

##### Distribution in Canada and Alaska.

NT, BC, AB, SK, MB, ON, QC, **NB**, NS (Bousquet and Laplante 2006).

### Tribe Histerini Gyllenhal, 1808

#### Atholus perplexus

(J. L. LeConte, 1863)

http://species-id.net/wiki/Atholus_perplexus

Map 11

##### Material examined. 

**New Brunswick, York Co.**, Charters Settlement, 45.8456°N, 66.7267°W, 10.VI.2010, R. P. Webster, beaver dam among sticks and debris on top of dam (2, RWC).

**Map 11.**
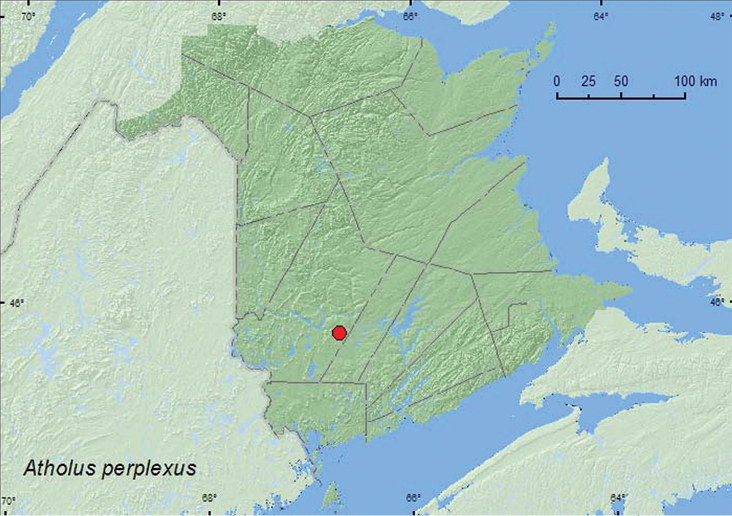
Collection localities in New Brunswick, Canada of *Atholus perplexus*.

##### Collection and habitat data.

*Atholus perplexus* has been collected from North American beaver (*Castor canadensis* Kuhl) lodges, muskrat (*Ondatra zibethicus* (L.)) nests, and in manure (Bousquet and Laplante 2006). The specimens from New Brunswick were collected among sticks and debris on top of a beaver dam. Beaver dung was present in the material on the top of the dam. The two adults were collected during June.

##### Distribution in Canada and Alaska.

SK, MB, ON, QC, **NB**, PE, NS (Bousquet and Laplante 2006).

#### Atholus sedecimstriatus

(Say, 1825)**

http://species-id.net/wiki/Atholus_sedecimstriatus

Map 12

##### Material examined. 

**New Brunswick, York Co.**, Charters Settlement, 45.8340°N, 66.7450°W, 20.VIII.2006, R. Webster, well decayed gilled and boletus mushrooms placed in an opening of 20 year-old regenerating mixed forest (1, RWC).

**Map 12.**
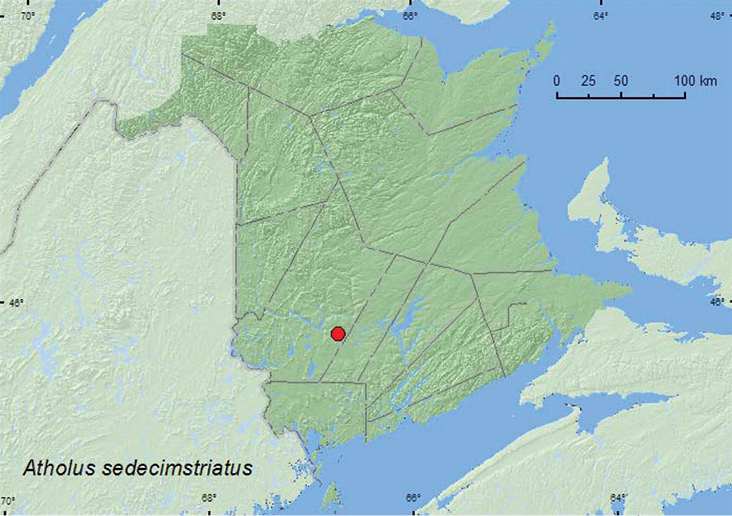
Collection localities in New Brunswick, Canada of *Atholus sedecimstriatus*.

##### Collection and habitat data.

This species occurs in compost, dung, and carrion (Bousquet and Laplante 2006). The specimen from New Brunswick was found in well-decayed mushrooms during August.

##### Distribution in Canada and Alaska.

ON, QC, **NB** (Bousquet and Laplante 2006).

#### Margarinotus cognatus

(J. E. LeConte, 1844)

http://species-id.net/wiki/Margarinotus_cognatus

Map 13

##### Material examined. 

**New Brunswick, Gloucester Co.**, 3 Vinot Rd. (Duguayville), 17.VI.1941, E. Dugway, 41-L68 (FIS) (1, AFC). **York Co.**, Charters Settlement, 45.8340°N, 66.7450°W, 8.VIII.2006, 14.VIII.2006, 20.VIII.2006, R. P. Webster, baited with well-decayed gilled and boletus mushrooms (7, RWC); 15 km W of Tracy off Rt. 645, 45.6848°N, 66.8821°W, 28.VI–7.VII.2009, R. Webster & M.-A. Giguère, old red pine forest, Lindgren funnel trap (1, AFC).

**Map 13.**
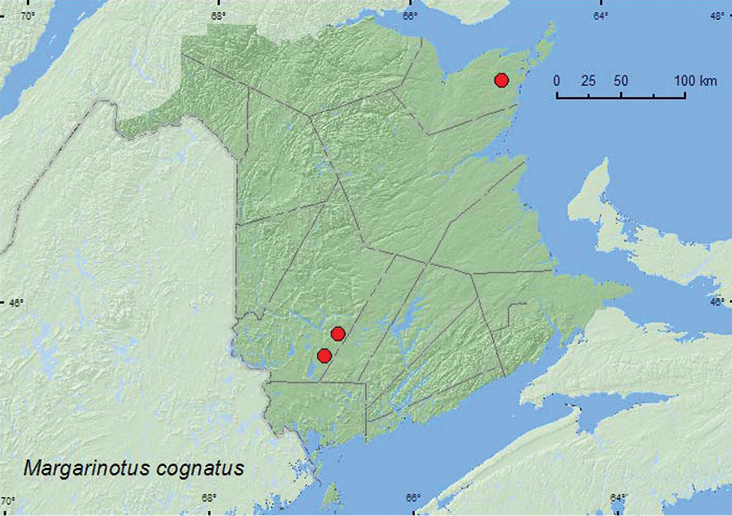
Collection localities in New Brunswick, Canada of *Margarinotus cognatus*.

##### Collection and habitat data.

Most adults from New Brunswick were collected from well-decayed gilled and boletus mushrooms that had been placed in an opening in a 20-year-old regenerating mixed forest. One individual was caught in a Lindgren funnel trap deployed in an old red pine forest. Adults were collected during June, July, and August. Little was previously known about the biology of this species (Bousquet and Laplante 2006).

##### Distribution in Canada and Alaska.

ON, QC, **NB**, NS (Bousquet and Laplante 2006).

#### Margarinotus confusus

Wenzel, 1944

http://species-id.net/wiki/Margarinotus_confusus

Map 14

##### Material examined. 

**New Brunswick, Carleton Co.,** Meduxnekeag Valley Nature Preserve, 46.1964°N, 67.6340°W, 31.V.2005, M.-A. Giguère & R. Webster, old mixed forest, in moist leaf litter at the margin of a vernal pond (1, RWC); Lower Woodstock, 46.1192°N, 67.5795°W, 7.V.2008, R. P. Webster, pasture, entrance to fox den (3, RWC). **York Co.**, Charters Settlement, 45.8430°N, 66.7275°W, 5.V.2006, R. P. Webster, in porcupine dung at the entrance of a porcupine den (1, RWC).

**Map 14.**
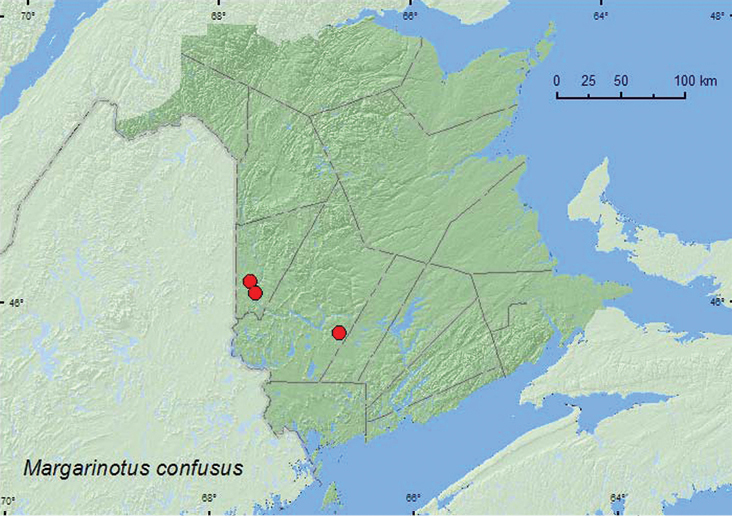
Collection localities in New Brunswick, Canada of *Margarinotus confusus*.

##### Collection and habitat data.

This species has been collected in woodchuck (*Marmota monax* (L.)) burrows and carrion (Bousquet and Laplante 2006). In New Brunswick, adults were collected from the entrance of a fox (*Vulpes* sp.) den, the entrance of a porcupine den in dung, and in moist leaf litter on the margin of a vernal pond. Adults were collected during May.

##### Distribution in Canada and Alaska.

ON, QC, **NB**, NS (Bousquet and Laplante 2006).

#### Margarinotus egregius

(Casey, 1916)

http://species-id.net/wiki/Margarinotus_egregius

Map 15

##### Material examined. 

**New Brunswick, Carleton Co.**, Lower Woodstock, 46.1192°N, 67.5795°W, 7.V.2008, R. P. Webster, pasture, entrance to fox den (1, RWC).

**Map 15.**
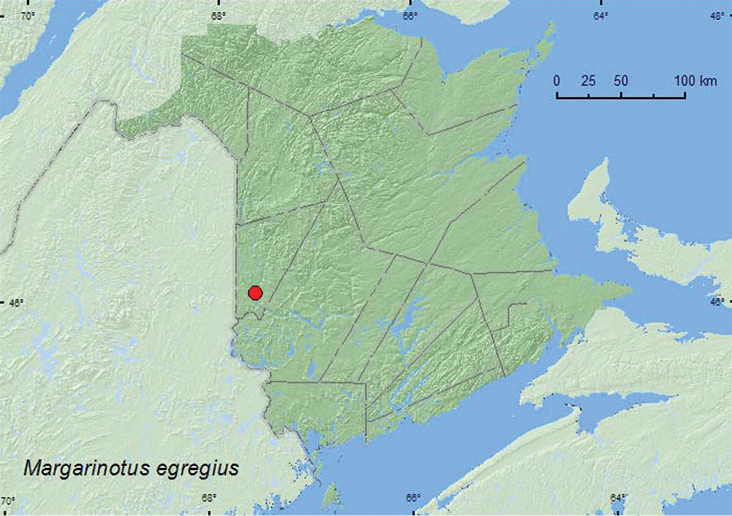
Collection localities in New Brunswick, Canada of *Margarinotus egregius*.

##### Collection and habitat data.

This species is commonly found in woodchuck burrows early in the spring and also in various decaying organic material such as carrion, dung, and decaying mushrooms (Bousquet and Laplante 2006). The specimen from New Brunswick was collected from the entrance of a fox den in May.

##### Distribution in Canada and Alaska.

MB, ON, QC, **NB**, NS (Bousquet and Laplante 2006).

#### Margarinotus harrisii

(Kirby, 1837)**

http://species-id.net/wiki/Margarinotus_harrisii

Map 16

##### Material examined. 

**New Brunswick, Charlotte Co.**, 3.0 km NW of Pomeroy Ridge, 45.3095°N, 67.4343°W, 16.VI.2008, R. P. Webster, old growth eastern white cedar swamp, in moss and leaf litter near small vernal pool (1, RWC). **Sunbury Co.**, Acadia Research Forest, 45.9866°N, 66.3841°W, 2-9.VI.2009, R. Webster & M.-A. Giguère, mature (110 year-old) red spruce forest with scattered red maple and balsam fir, Lindgren funnel trap (1, RWC).

**Map 16.**
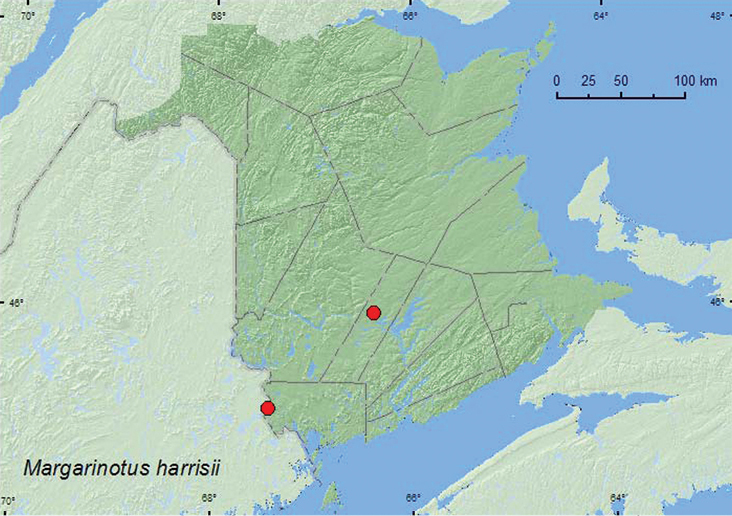
Collection localities in New Brunswick, Canada of *Margarinotus harrisii*.

##### Collection and habitat data.

One of the New Brunswick specimens was collected in moss and leaf litter near a vernal pond, the other from a Lindgren funnel trap. In Indiana (USA), the species was usually found in cow dung and was frequent under “chunks” on the beach of Lake Michigan (Blatchley 1910). Adults were collected during June in New Brunswick.

##### Distribution in Canada and Alaska.

BC, AB, SK, MB, ON, QC, **NB** (Bousquet and Laplante 2006).

#### Margarinotus merdarius

(Hoffman, 1803)

http://species-id.net/wiki/Margarinotus_merdarius

Map 17

##### Material examined. 

**New Brunswick, Kings Co.**, *ca*. 2 km WSW of Browns Flat, 45.4667°N, 66.1668°W, 8.VII.2009, S. Makepeace & R. Webster, in barred owl nest box, moist organic debris and sawdust with owl pellets, small bones, feathers, with urine smell (1, RWC). **Queens Co.**, Central Hampstead, 45.6575°N, 66.1412°W, 13.VII.2006, S. Makepeace & R. Webster, hardwood ridge, in barred owl nest in tree hole (1, RWC); Elm Hill, 45.7140°N, 66.1315°W, 27.VI.2007, S. Makepeace & R. Webster, in barred owl nest in tree hole in red oak, damp (urine smell) organic material with feathers, fur and small bones (1, RWC); Cumberland Bay, 46.0000°N, 65.9333°W, 28.VI.2009, Makepeace & R. Webster, in barred owl nest, moist leaves and debris with owl pellets, small bones, with urine smell (1, RWC); *ca*. 1.5 km NW of McAlpines, 45.7333°N, 66.1333°W, 8.VII.2009, S. Makepeace & R. Webster, in barred owl nest box, moist organic debris and sawdust with owl pellets, small bones, feathers, with urine smell (2, RWC). **York Co.**, Marysville, 45.9750°N, 66.5700°W, 22.VI.2007, S. Makepeace & R. Webster, nest box contents of barred owl, with dry organic material and remains of squirrel, birds, and insect parts (1, NBM).

**Map 17.**
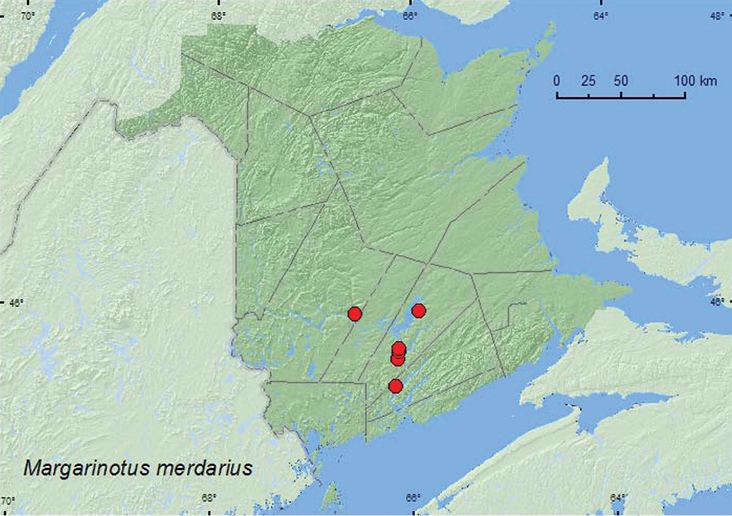
Collection localities in New Brunswick, Canada of *Margarinotus merdarius*.

##### Collection and habitat data.

In Europe, this species occurs in dung, fungi, compost, and decaying vegetables and in bird nests and henhouses (Vienna 1980). All specimens of this adventive species from New Brunswick were collected from nest material from barred owl nests (most with chicks) in natural cavities in trees or in artificial nest boxes. Adults were captured during June and July.

##### Distribution in Canada and Alaska.

BC, AB, MB, ON, QC, **NB**, NS (Bousquet and Laplante 2006).

#### Margarinotus stygicus

(J. E. LeConte, 1845)

http://species-id.net/wiki/Margarinotus_stygicus

Map 18

##### Material examined. 

**New Brunswick, York Co.**, Charters Settlement, 45.8395°N, 66.7391°W, 19.VI.2004, R. P. Webster, pitfall trap baited with dog dung (1, RWC).

**Map 18.**
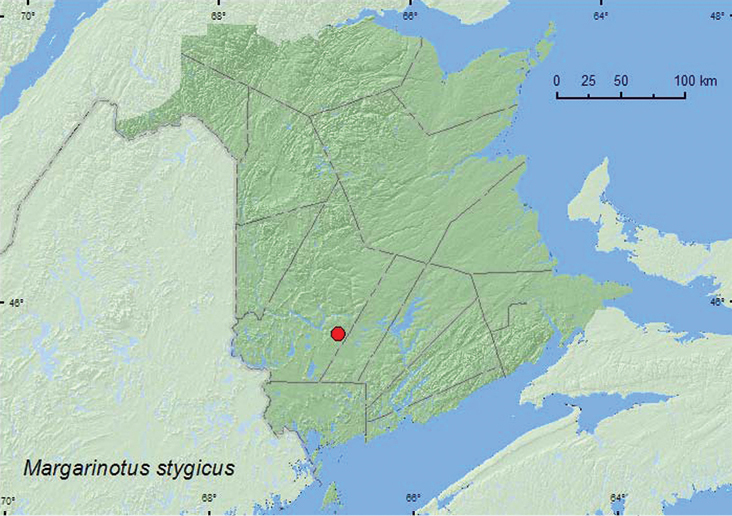
Collection localities in New Brunswick, Canada of *Margarinotus stygicus*.

##### Collection and habitat data.

Relatively little is known about the habitat requirements of this species. The scant habitat data available suggest that the species is probably associated with mammal nests. Adults have been collected by sifting around a deserted mouse nest (Blatchley 1910) and from a fox burrow (Bousquet and Laplante 2006). The specimen from New Brunswick was captured during June in a pitfall trap baited with dog dung.

##### Distribution in Canada and Alaska.

MB, ON, **NB**, NS (Bousquet and Laplante 2006). Majka (2008) considered this species to be disjunct in Nova Scotia due to a lack of records from Quebec and other regions of Atlantic Canada.

## Supplementary Material

XML Treatment for Acritus exiguus

XML Treatment for Euspilotus rossi

XML Treatment for Gnathoncus barbatus

XML Treatment for Gnathoncus communis

XML Treatment for Hypocaccus fitchi

XML Treatment for Dendrophilus kiteleyi

XML Treatment for Dendrophilus punctatus

XML Treatment for Platysoma cylindricum

XML Treatment for Platysoma deficiens

XML Treatment for Platysoma leconti

XML Treatment for Atholus perplexus

XML Treatment for Atholus sedecimstriatus

XML Treatment for Margarinotus cognatus

XML Treatment for Margarinotus confusus

XML Treatment for Margarinotus egregius

XML Treatment for Margarinotus harrisii

XML Treatment for Margarinotus merdarius

XML Treatment for Margarinotus stygicus
